# Progressive multifocal leukoencephalopathy in a lung transplant recipient

**DOI:** 10.1002/ccr3.8626

**Published:** 2024-03-08

**Authors:** Jason Sakizadeh, Michael J. Davis, Lauren Fontana

**Affiliations:** ^1^ University of Minnesota Medical School Twin Cities Campus Minneapolis MN USA; ^2^ Division of Infectious Diseases and International Medicine University of Minnesota Medical School Twin Cities Campus, Infectious Disease Minneapolis MN USA

**Keywords:** case report, progressive multifocal leukoencephalopathy, JC virus, lung transplant, immunocompromised

## Abstract

Progressive multifocal leukoencephalopathy (PML) is a rare and fatal demyelinating disease of the central nervous system (CNS). The case we describe highlights the importance of considering a diagnosis of PML early (<1 year) after lung transplant.

## 1 INTRODUCTION

PML was first characterized in 1958 as a severe demyelinating infectious process caused by the reactivation of the polyomavirus known as JC virus.^1^, ^2^ Typically in immunocompetent hosts, the virus remains dormant in kidneys, bone marrow, and lymphoid tissue.^1^ However, in the setting of an immunocompromised host, the virus can infect and destroy the myelin‐producing cells in the CNS leading to the rare demyelinating disease PML.^1^

PML can occur at any point following solid organ transplant due to the immunocompromised state with the median time after transplant estimated to be 17 months (range less than 1 month to 20 years).^3^ Comparatively, the incidence of PML is highest in kidney transplant recipients and lowest in lung and heart transplant recipients.^3^ In lung and heart transplant recipients, one retrospective cohort study determined the risk of PML as 1.24 per 1000 post transplantation years.^3^ Here, we describe the case of a lung transplant recipient who developed PML 7 months post‐transplant. We also provide a literature review of the previous PML cases reported in lung transplant recipients.

## 2 CASE HISTORY/EXAMINATION

A 69‐year old man with a history of end‐stage chronic obstructive pulmonary disease (COPD) with pulmonary hypertension underwent bilateral lung transplantation. Induction immunosuppression included basiliximab and high‐dose methylprednisolone followed by maintenance immunosuppression with tacrolimus, mycophenolate mofetil, and prednisone. Cytomegalovirus (CMV) donor and recipient statuses were negative and positive, respectively. Epstein–Barr virus (EBV) was positive for both the donor and the recipient. Antimicrobial prophylaxis included trimethoprim‐sulfamethoxazole for *Pneumocystis jirovecii* pneumonia (PJP), oral nystatin for candidiasis, and an anticipated 3 months of valganciclovir for CMV. He was discharged on Day 16 post‐transplant.

Seven months post‐transplant, he developed lethargy, gait instability, delayed speech, and progressively worsening pulmonary function tests (PFTs). His worsening PFTs raised concern for acute cellular rejection (ACR) prompting a diagnostic bronchoscopy with bronchoalveolar lavage (BAL). After the BAL was completed, he was started on empiric treatment for ACR with intravenous methylprednisolone 1 g daily for 3 days followed by a 10‐day prednisone taper which was tapered down to his maintenance dose of 7.5 mg daily. The BAL was non‐diagnostic of ACR. The patient progressively developed short‐term memory loss, behavior changes, episodes of staring into space with confusion, and more limited verbal responses over the course of 2–3 weeks, with acute worsening of gait and aphasia approximately 3 weeks after the initiation of empiric steroids.

Neurological examination was notable for inattention, delayed response to questions, intermittent following of commands, tremors, and a few beats of clonus bilaterally. He had difficulty following the full motor examination, but strength was noted to be five out of five with elbow flexion, elbow extension, and hand grip bilaterally. He was moving bilateral lower extremities antigravity. Gait examination was deferred. He was subsequently admitted to the hospital for further evaluation of worsening neurologic deficits.

## 3 INVESTIGATIONS, TREATMENT, AND DIFFERENTIAL DIAGNOSIS

**FIGURE 1:**
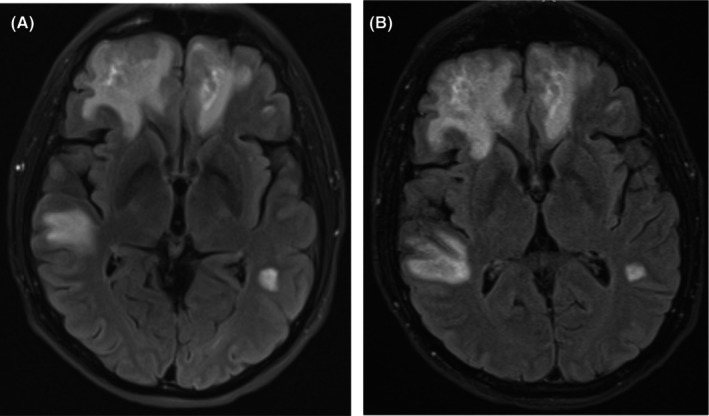
Slices from T2‐weighted fluid‐attenuated inversion recovery (FLAIR) sequences from MRI brain from hospital Day 1 (A) and hospital Day 13 (B) demonstrating abnormal white matter signals bilaterally.

Computed tomography (CT) of the head demonstrated bifrontal, right greater than left temporal deep and subcortical white matter hypoattenuation which was thought to represent a subacute white matter process. Brain magnetic resonance imaging (MRI) with and without contrast revealed abnormal white matter signals throughout the bilateral frontal, parietal, and temporal lobes (predominantly in the right frontal lobe; Figure 1A). Lumbar puncture was performed on hospital Day 2. Cerebrospinal fluid (CSF) showed a nucleated cell count of 2/μL, glucose of 52 mg/dL (blood glucose was 80 mg/dL on the day of lumbar puncture; the CSF/blood glucose ratio was 0.65), protein of 74.9 mg/dL. CSF cytology, *Toxoplasma gondii* PCR, EBV PCR, herpes simplex virus (HSV) 1 and 2 PCR, and venereal disease research laboratory (VDRL) tests were negative. The CSF JC virus PCR was positive with 4100 copies/mL. The plasma JC virus PCR (collected hospital Day 8) was positive with 3500 copies/mL (plasma and CSF JC virus PCR tests were performed at Eurofins Viracor laboratory in Lenexa, KS). Tacrolimus level was 11.1 μg/L (tacrolimus goal was 8–12 μg/L). Serum white blood cell (WBC) count nadir was 1.2 × 10e3/μL on hospital Day 9 with an absolute neutrophil count (ANC) of 0.7 × 10e3/μL and absolute lymphocyte count (ALC) of 0.3 × 10e3/μL. Immunoglobulin G (IgG) level was 464 mg/dL. Repeat brain MRI with and without contrast on hospital Day 13 showed progression of the bilateral white matter lesions (Figure 1B). Repeat lumbar puncture on hospital Day 15 was obtained for further evaluation of new fevers with a repeat JC virus quantitative PCR demonstrated an increase in the CSF viral load at 8000 copies/mL. Plasma JC PCR was not repeated.

Given the concern for tacrolimus neurotoxicity, the tacrolimus was held and he was given basiliximab. To mitigate the leukopenia, trimethoprim‐sulfamethoxazole and valganciclovir doses were reduced, the mycophenolate mofetil was stopped, and granulocyte colony stimulating factor was prescribed. He also received intravenous immunoglobulin for hypogammaglobulinemia.

Our initial differential diagnosis included calcineurin inhibitor‐induced toxic leukoencephalopathy which is type of posterior reversible encephalopathy syndrome (PRES), post‐transplant lymphoproliferative disease, opportunistic CNS infection such as PML or toxoplasmosis, viral encephalopathy, and stroke. The constellation of findings including rising CSF JC viral loads, radiographic imaging, and neurologic changes made PML secondary to JC virus the most unifying diagnosis.

## 4 OUTCOMES AND FOLLOW‐UP

The patient's mentation and speech gradually worsened throughout the hospitalization, and he was ultimately discharged with hospice on hospital Day 25. The patient expired 3 days after discharge.

## 5 DISCUSSION

As of this publication, there have been 11 reported cases of PML secondary to JC virus after lung transplantation, one reported case secondary to BK polyomavirus virus after lung transplantation, and one suspected case of PML after lung transplantation that could not be confirmed.^3^, ^4^, ^5^, ^6^, ^7^, ^8^, ^9^, ^10^, ^11^, ^12^, ^13^ Table 1 outlines patient characteristics of the 13 cases in addition to the case of the patient detailed in this report. The patient described in this case was the oldest at time of lung transplant at 69 years‐old (range of ages in prior cases was 38–66).^3^, ^4^, ^5^, ^6^, ^7^, ^8^, ^9^, ^10^, ^11^, ^12^, ^13^ The case is also unique as the patient developed symptoms of PML within a year after transplant, which is contrary to 11 of the 13 reported cases where symptoms developed at least 1 year after transplant (range 1 year to 63 months).^3^, ^4^, ^5^, ^6^, ^7^, ^8^, ^9^, ^10^, ^11^, ^12^, ^13^

**TABLE 1:** Characteristics of reported PML cases in lung transplant recipients.

Case report author and year	Reason for lung transplantation	Age at time of lung transplant (years)	Time from transplant to development of PML symptoms	Induction IS	Maintenance IS	Rejection reported in the case?	Rejection treatment	Outcome^a^
Ouens et al., 2000^4^	Bronchiectasis	43	15 months	RATG	Cyclosporine, azathioprine, prednisone	Yes (x3)	MP	Death at 15 months
Shitrit et al., 2003^5^	IPF	55	7 months	Not specified	Tacrolimus, MMF, prednisone	No	Not applicable	Stable at 7 months
Waggoner et al., 2009^6^	IPF	38	18 months	MP	Tacrolimus, azathioprine, prednisone	Yes (x7)	MP (x4), RATG (x2), alemtuzumab (X1)	Death at 4 months
Mateen et al., 2011^3^	COPD	62	27 months	Not specified	Not specified	No	Not applicable	Death at 16 months
Mateen et al., 2011^3^	IPF	39	42 months	Not specified	Not specified	No	Not applicable	Death at 51 months
Mateen et al., 2011^3^	Histiocytosis X	47	63 months	Not specified	Not specified	No	Not applicable	Death at 64 months
Lobo et al., 2013^7^	IPF	61	14 months	Basiliximab	Cyclosporine, MMF, prednisone	Yes	Rituximab and intravenous IgG	Death at 2 weeks
Moua et al., 2013^8^	Chronic HP	61	5 months	None	Tacrolimus, prednisone	Yes	Not specified	Death at 6 months
Panchabhai et al., 2016^9^	COPD	60	16 months	Not specified	Tacrolimus, prednisone	No	Not applicable	Death at 3 weeks
Hamad et al., 2017^10^	Pulmonary fibrosis related to MCTD	55	14 months	Basiliximab	Tacrolimus, MMF, prednisone	No	Not applicable	Stable at 7 months
Ishii et al., 2019^11^	Pulmonary LAM	55	60 months	Not specified	Tacrolimus, MMF, prednisone	No	Not applicable	Death at 3 months
Crowhurst et al., 2020^12^	Chronic HP	66	19 months	MP	Tacrolimus, MMF, prednisolone	Yes	MP	Death at 3 months
Chatterton et al., 2022^13^	RA‐associated ILD	64	1 year	Not specified	Not specified	No	Not applicable	Stable at 8 months
Current Case	COPD	69	7 months	Basiliximab	Tacrolimus, MMF, prednisone	Yes	MP	Death at 2 months

Abbreviations: COPD, chronic obstructive pulmonary disease; HP, hypersensitivity pneumonitis; ILD, interstitial lung disease; IPF, idiopathic pulmonary fibrosis; IS, immunosuppression; LAM, lymphangioleiomyomatosis; MCTD, mixed connective tissue disease; MMF, mycophenolate mofetil; MP, methylprednisolone; PML, progressive multifocal leukoencephalopathy; RA, rheumatoid arthritis; RATG, rabbit anti‐thymocyte globulin.

^a^ Outcome after symptom onset and/or diagnosis of PML.

Although rare, PML should be considered in the differential diagnosis in an immunocompromised patient who develops new neurologic deficits over weeks to months. Clinically, patients can develop a range of neurological deficits depending on the part of the brain affected including deficits in motor function, vision, cognition, and speech.^14^, ^15^ An MRI of the brain will characteristically show hyperintense multifocal white matter brain lesions at any part of the brain on T2‐weighted images.^14^ Although CSF studies can be non‐specific in PML with normal cell counts, detection of JC virus in the right clinical context can be diagnostic.^14^ A brain biopsy is not without risks but is considered in certain circumstances when the diagnosis is unclear. If diagnostic, brain biopsy will show demyelinating lesions and atypical glial cells.^1^

The diagnosis in this case was confirmed with JC virus CSF viral titers. Because there are numerous diagnoses on the differential to consider in an immunosuppressed post‐transplant patient with white matter changes on brain imaging, it is important to consider PML and include JC virus PCR in initial CSF studies in this patient population. This is the second reported case of PML in lung transplant recipients to demonstrate increasing JC viral DNA loads on subsequent PCR tests of CSF.^13^ Positive JC virus PCR of CSF has been shown to have a specificity of 95.8% for PML, albeit the majority of patients in the study had advanced HIV infections and were not organ transplant recipients.^16^ Furthermore, when considering JC virus PCR of CSF, it is important to obtain a quantitative viral load rather than just a qualitative value as it can increase the positive predictive value of the test from 10.4% to 100.0% (≥2.7 log copies/mL is strongly indicative of PML).^17^ In this case, the patient's initial CSF JC viral load was 4100 copies/mL (3.6 log copies/mL) on hospital Day 2 and 8000 copies/mL (3.9 log copies/mL) on hospital Day 15, which would be considered strongly indicative of PML especially with the increasing viral load on a subsequent CSF evaluation. Additionally, a study of patients infected with HIV with PML showed that JC viral DNA loads >3.64 log copies/mL in CSF correlated significantly with shorter survival in patients not receiving highly active antiretroviral therapy, which suggests that JC viral DNA loads could act as a prognosticating tool as well.^18^

In the context of lung transplant recipients, it is likely the combination of the underlying immunosuppression rather than any one immunosuppressive agent itself that increases the risk of PML. Immunosuppressive drugs can be classified based on their risk of predisposing patients to PML.^19^ Our patient received basiliximab and methylprednisolone for induction immunosuppression followed by tacrolimus, mycophenolate mofetil, and prednisone for maintenance immunosuppression. Two of the 13 cases previously described also specified basiliximab as part of their induction immunosuppression regimen (Table 1).^7^, ^10^ While there are limited data in the literature regarding the risk of PML associated with basiliximab, one analysis of the World Health Organization adverse drug reaction database provided evidence for a potential association between basiliximab and PML.^20^ Additional research is needed to understand if there is a defined association.

The maintenance immunosuppression regimen was described in nine of the thirteen previously described cases, with all nine receiving prednisone or prednisolone and a calcineurin inhibitor (seven received tacrolimus, two received cyclosporine; Table 1).^4^, ^5^, ^6^, ^7^, ^8^, ^9^, ^10^, ^11^, ^12^ In addition, five of the nine received a three‐drug regimen with mycophenolate mofetil, and two received a three‐drug regimen with azathioprine.^4^, ^5^, ^6^, ^7^, ^8^, ^9^, ^10^, ^11^, ^12^ Tacrolimus, cyclosporine, mycophenolate mofetil, and azathioprine are less likely to be associated with PML when used as monotherapy, but the risk is higher when used in combination with other agents that also predispose one to PML.^19^

In post‐lung transplant patients with PML, immunosuppressive agents are often modified in an effort to limit PML disease progression, but decreasing the doses must be balanced with the risk of rejection. Immunosuppression modulation was undertaken as a treatment measure in at least 11 of the 14 cases (including this case) of PML in post‐lung transplant patients (treatment was not reported in three of the cases).^3^, ^4^, ^5^, ^6^, ^7^, ^8^, ^9^, ^10^, ^11^, ^12^, ^13^

There were limitations to the literature review portion of the case discussion. For one, not all reported cases of PML after lung transplant specified the induction and maintenance immunosuppression regimens or the transplant rejection history and treatment. The incomplete data from the literature restricted our ability to identify potential trends in the clinical variables between cases.

## 6 CONCLUSION

Overall, PML should be suspected in immunocompromised patients including lung transplant recipients presenting with subacute neurological deficits. It is an important diagnosis to consider early on in the post‐transplant course (<1 year) if the clinical syndrome and radiographic features are consistent with PML. Brain MRI can show white matter changes that should make one consider a PML diagnosis, and quantitative JC viral PCR values from CSF can be valuable diagnostic tools with potential value in prognostication as well.

## AUTHOR CONTRIBUTIONS

**Jason Sakizadeh:** Writing – original draft. **Michael J. Davis:** Writing – review and editing. **Lauren Fontana:** Writing – review and editing.

## FUNDING INFORMATION

None.

## CONFLICT OF INTEREST STATEMENT

The authors (Jason Sakizadeh, Michael J. Davis, and Lauren Fontana) have no conflicts of interest that are directly relevant to this case report.

## CONSENT

Written informed consent was obtained from the patient to publish this report in accordance with the journal's patient consent policy.

## Data Availability

Data sharing is not applicable to this article as no new data were created or analyzed in this study.
